# A Rare Case of Richter Transformation to Both Clonally Unrelated and Clonally Related Diffuse Large B-Cell Lymphoma in the Same Patient

**DOI:** 10.1155/2024/7913296

**Published:** 2024-08-31

**Authors:** Michelle D. Don, Carlos Casiano, Huan-You Wang, Mikhail Gorbounov, Wei Song, Edward D. Ball

**Affiliations:** ^1^ Division of Laboratory and Genomic Medicine Department of Pathology University of California San Diego, 3855 Health Sciences Drive Room 3074, La Jolla, San Diego 92093, CA, USA; ^2^ Department of Pathology Loma Linda University Health, 11370 Anderson St, Suite 2950, Loma Linda 92354, CA, USA; ^3^ Division of Blood and Marrow Transplant Department of Medicine University of California San Diego, 3855 Health Sciences Drive, La Jolla, San Diego 92093, CA, USA

## Abstract

Richter transformation (RT) is a rare sequelae of chronic lymphocytic leukemia (CLL)/small lymphocytic lymphoma (SLL). The clonal relationship of the RT to the underlined CLL/SLL is an important prognostic factor as clonally related RT has a worse prognosis than that of clonally unrelated RT. The development of more than one RT in the same patient is exceedingly rare and prior reports have shown cases consisting of RT to diffuse large B-cell lymphoma (DLBCL) and a subsequent or synchronous Hodgkin lymphoma. Here, we present a rare case of RT first to a clonally unrelated DLBCL and subsequently a clonally related DLBCL. Additionally, we retrospectively conducted next-generation sequencing studies of both RT's and found different mutational landscapes, including more clinically aggressive mutations identified in the clonally related RT. To our knowledge, this is the first reported case of clonally related and clonally unrelated RT, both of which are DLBCL, in the same patient.

## 1. Introduction

Richter transformation (RT) is a rare sequelae in the setting of chronic lymphocytic leukemia (CLL)/small lymphocytic lymphoma (SLL), with an annual incidence rate around 0.5% per year for all CLL/SLL patients [1] and with an overall poor prognosis [2]. At the histopathologic level, RT can be assigned to the most common variant of diffuse large B-cell lymphoma (DLBCL) and the much less variant of classic Hodgkin lymphoma (CHL) [3]. On the molecular front, RT can be divided into clonally related to the underlying CLL/SLL, which is the most common, occurring approximately in 80% of cases, and clonally unrelated, which is less likely and occurs in approximately 20% of cases [3]. The clonal relationship of the RT to the underlying CLL/SLL is important to prognosis, as clonally unrelated RT has a prognosis similar to that of a *de novo* DLBCL, and clonally related RT has a more dismal prognosis [4–6]. While RT is rare, the development of two RTs within the same patient, either subsequent or synchronous, is exceedingly rare. To our knowledge, all cases of two RT, occurring within the same patient, where one is clonally related and one is clonally unrelated, have been DLBCL and CHL [7–10]. Here, we describe a case of longstanding CLL/SLL with RT to a clonally unrelated DLBCL, followed by a clonally related DLBCL. Additionally, we retrospectively performed next generation sequencing (NGS) on each of the clonally unrelated and clonally related RT cases and compared them to a prior NGS study on the patient's CLL/SLL to further investigate the clonal progeny of each of the RT's.

## 2. Case Presentation

A 70-year-old male with a longstanding history of CLL was initially diagnosed in 2005 at an outside hospital and partially transferred care to our institution in 2013. Prior treatment included six cycles of bendamustine and rituximab (BR). Upon establishing care in 2013 at our hospital, a bone marrow biopsy showed 50% involvement by CLL, consisting of small lymphocytes with no evidence for a RT. The immunophenotype by flow cytometry of the bone marrow aspirate at that time showed the leukemic cells were positive for CD5, CD19, CD20 (partial and dim), CD22, CD23, CD38, CD43, CD45, CD79b (partial and dim), surface lambda light chain; they were negative for CD3, CD10, CD81, FMC-7 and surface kappa light chain. Cytogenetics at that time showed an abnormal and complex karyotype, 46, XY, del(6) (q23), del(11) (q21q23), del(13) (a14q31) (cp4)/45, XY, del(3) (q??26), der(4)add(4) (p15add) (4) (q?25), del(6) (q23), −9, del(11) (q21q23)[7]/46, XY[12]. Fluorescence in situ hybridization (FISH) studies showed deletion 11q. The patient had additional treatments with BR and CXCR4 antagonist followed by ibrutinib. In 2016, the patient showed clinical signs of progression with rapidly rising white blood cell counts, progressive lymphadenopathy, and tumor lysis syndrome; however, no tissue biopsy was obtained at that time. Due to clinical concern for RT, treatment with rituximab, etoposide, prednisone, vincristine, cyclophosphamide, and doxorubicin (R-EPOCH), was initiated at an outside hospital. Upon returning to our institution shortly after, venetoclax was initiated. The patient continued to have persistent CLL and in September of 2018 was treated with anti-CD19 chimeric antigen receptor (CAR)-T cell therapy, and by October of 2018 there was no morphologic or immunophenotypic evidence for CLL. In January of 2020 the patient was diagnosed with a therapy-related myeloid neoplasm in the form of a low-grade myelodysplastic neoplasm (MDS) with deletion 20q identified by karyotype and FISH but with no morphologic, immunophenotypic, or genetic evidence for CLL.

**Figure 1:**
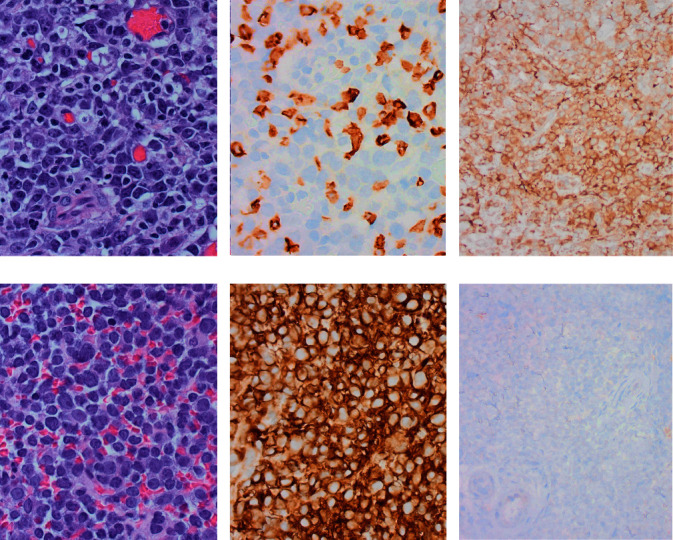
Comparison of two Richter transformations (RT) to diffuse large B-cell lymphoma (DLBCL) within the same patient. H&E-stained sections of a jejunal perforation showing increased medium to large atypical lymphoid cells (a) that were notably negative for CD5 (b) and positive for CD10 (c), found to be DLBCL RT to a clonally unrelated chronic lymphocytic leukemia/small lymphocytic lymphoma (CLL/SLL). A biopsy from a retroperitoneal mass in the same patient, 7 months later, also showed increased medium to large atypical lymphoid cells (d), but these cells are positive for CD5 (e) and negative for CD10 (f), found to be a clonally related DLBCL RT to the patient's CLL/SLL. ((a), (b), (d), (e) ×40 objective; (c) and (f) ×20 objective).

In August of 2021, the patient presented with abdominal pain and CT scan showing a large mass-like retroperitoneal nodal conglomerate and small bowel obstruction. A retroperitoneal lymph node excisional biopsy at that time showed sheets of small lymphocytes with an immunophenotype similar to that described above (bone marrow biopsy from 2013). A diagnosis of SLL with no evidence for RT was rendered. The following day, after the lymph node biopsy, the patient underwent exploratory laparotomy with small bowel resection due to jejunal perforation resulting from the small bowel obstruction, which showed DLBCL with germinal center (GC) phenotype (Figures 1(a), 1(b), 1(c)). FISH studies showed *MYC* gene and immunoglobulin heavy (*IGH*) chain gene rearrangements, with no evidence for *BCL2* or *BCL6* gene rearrangements. Given the history of CLL/SLL and the presence of the GC phenotype, there was speculation that this lymphoma was clonally unrelated to the known CLL/SLL. To further investigate, molecular studies for *IGH* and immunoglobulin kappa (*IGK*) gene rearrangements were performed, which showed a monoclonal rearrangement of both the *IGH* and *IGK* genes. These findings were compared to *IGH* and *IGK* gene rearrangement studies from a former CLL/SLL specimen from 2018 and there were no similarities between them (Table 1), thus consistent with a clonally unrelated RT, and for the purposes of this report, will be referred to as RT-1. The patient was further treated with rituximab, cyclophosphamide, doxorubicin, and vincristine (R-CHOP) and palliative radiation to the retroperitoneum. Approximately 7 months later, in March of 2022, a biopsy of the retroperitoneal mass-like conglomerate was again performed, and showed DLBCL with a non-GC phenotype (Figures 1(d), 1(e), and 1(f)). In this biopsy specimen, the immunophenotype more closely resembled that of the patients prior CLL/SLL. B-cell clonality studies were again performed and compared to the same prior to CLL/SLL specimen, which showed similar patterns (Table 1), consistent with a clonally related RT, and for the purposes of this paper will be referred to as RT-2. Unfortunately, the patient was not a candidate for further treatment and expired shortly after.

In order to evaluate the mutational profile between different specimens, retrospective mutation profiling studies, using our in-house 123 gene NGS panel of common gene mutations identified in hematopoietic neoplasms, were performed on both of the clonally unrelated and clonally related RT specimens and compared to a prior NGS study from a bone marrow specimen in 2018, which showed only CLL without RT (Table 2). The RT-1 specimen showed three pathogenic mutations and the RT-2 specimen showed seven pathogenic mutations, most of which were previously reported driver mutations [11]. However, no same mutations were identified between the two RT, further confirming two independent transforming processes. The 2018 CLL bone marrow case showed a single *SF3B1* H662Y mutation. To confirm the *SF3B1* mutation was related to the CLL and not the patient's MDS, an NGS report was reviewed from a bone marrow biopsy from December 2018 in which MDS was present with no evidence of CLL. This showed no clinically significant somatic variants detected in the regions interrogated (data not shown), indicating the *SF3B1* mutation was associated with patient's CLL.

## 3. Discussion

We presented a complex and interesting case of longstanding CLL/SLL that went into remission for three years and then showed RT. RT-1 occurred first in the form of DLBCL with GC phenotype. Within 7 months from RT-1, a second RT, RT-2, was diagnosed as DLBCL but with non-GC phenotype. Immunophenotypic features and molecular B-cell clonality studies for *IGH* and *IGK* gene rearrangements were performed and compared to a prior CLL/SLL specimen for the patient. RT-1 showed no definitive evidence for a clonal relationship to the original CLL/SLL, but RT-2 showed evidence for a clonal relationship. In addition, mutation profiles from the two RT specimens showed no overlapping mutations, further supporting the notion that the two RTs resulted from distinct processes.

RT is a rare sequelae of CLL/SLL, and two RT's within the same patient are exceedingly rare. The prognosis of DLBCL RT can be variable, but is overall considered poor [12]. RT-1 was a clonally unrelated DLBCL, which should bear a prognosis more similar to *de novo* DLBCL, and treated accordingly. Clonally related DLBCL RT has a shorter median survival compared with clonally unrelated DLBCL RT [4]. Thus, it is clinically relevant for prognostic and treatment purposes to further investigate the clonal relationship of a DLBCL RT [13]. The best way to assess clonal relationship of RT is by DNA Sanger sequencing (SS) allowing for identification and comparison of the major IGHV clone. SS was not performed in this study; however, immunophenotype was utilized as the first insight into the potential clonal relationship, followed by polymerase chain reaction (PCR)-based methods to assess for rearrangements of *IGH* and *IGK* genes with comparisons of the specimen. Another potential method of assessing clonal relationship, however not utilized in this study, is described in Broséus et al., where the authors characterized a CLL epigenetic imprint that can be used to assess clonal relationship of an RT sample even without the initial CLL counterpart [5]. Overall, the importance of obtaining a full immunophenotype in cases of RT, as a first insight into clonal relatedness, continues to be of significant value.

Retrospective NGS studies were performed to further investigate the mutational landscape of patient's original CLL/SLL as compared to both RT-1 and RT-2. The original CLL/SLL was found to harbor only a *SF3B1* gene mutation, a predictor of poor clinical outcome in CLL/SLL cases [14]. A large multi-institutional study of over 600 CLL cases found that *SF3B1* mutations showed no impact on progression to RT [15]. The persistence of *SF3B1* mutation in RT-2, at least supports the clonal relationship of RT-2 to the patient's CLL/SLL.

The molecular profiles of DLBCL RT are thought to be heterogeneous without overlap to *de novo* DLBCL cases [5, 16, 17]. Multiple previously reported CLL driver mutations were identified in RT-2 including *XPO1, POT1, KRAS,* and *TP53* [11], which have been shown to be more commonly mutated in RT when compared to CLL/SLL [18]. Additionally, when compared to the mutational profile of RT-1, RT-2 harbors some mutations that are considered more clinically aggressive including the acquisition of a *TP53* mutation, which is associated with more aggressive disease and chemoresistance [19]. The clonally unrelated RT, RT-1 which harbors a GC phenotype, showed the presence of *MYD88*, a CLL driver mutation, as well as mutations in genes implicated in cancer pathogenesis, *TET2* and *KMT2D*, which have also been seen in RT [18]. *MYD88* mutations, specifically *MYD88* L265P mutation, are significantly associated with activated B-cell-like (ABC) DLBCL [20–22]. Although rare, *MYD88* mutations can also be seen in DLBCL with GC phenotype [21]. In this case, RT-1 showed a *MYD88* S219C mutation, a rare variant that has not been shown to have preference for a specific cell of origin [23]. The presence of *MYD88* mutation in DLBCL is associated with a poor outcome [21, 22].

The development of two RT's within the same patient could be due to a variety of factors including the known heterogeneous nature of CLL/SLL, which includes the proliferation of CLL/SLL cub-clones [6, 24]. Additionally, prior extensive treatment and the microenvironment likely played a role in this patient's progression [25]. The immune microenvironment of RT has been shown have higher PD-L1 expression amongst histiocytes and dendritic cells and higher PD-1 expression in the large B-cell of RT as compared to CLL, indicating that the microenvironment of CLL and RT are different [26]. PD-1 expression in *de novo* DLBCL has also been shown to be weak compared to that of RT [27]. Additionally, PD-1 expression by large B-cell in RT highly correlates to a clonally related RT [28]. Thus, it is postulated that the PD-1/PD-L1 axis for RT-1 and RT-2 in this patient is likely different.

The exact mechanisms that drive CLL transformation to RT are not entirely understood let alone transformation two both a clonally unrelated and clonally related RT in the same patient.

While RT to clonally related DLBCL has been studied, there is minimal but growing knowledge regarding clonally unrelated RT [29]. Furthermore, insight into development of both clonally related and clonally unrelated RT within the same patient is scarce.

## 4. Conclusion

We presented a patient with a long and complex history of CLL/SLL who developed RT to a clonally unrelated DLBCL and then a clonally related DLBCL, which is an exceedingly rare occurrence. To our knowledge, this is the first reported case.

## Figures and Tables

**Table 1:** Comparison of immunoglobulin heavy chain and immunoglobulin kappa gene rearrangement results between chronic lymphocytic leukemia/small lymphocytic lymphoma, clonally unrelated Richter transformation, and clonally related Richter transformation within the same patient.

Case	IgH fragment 1 (bP)	IgH fragment 2 (bP)	IgH fragment 3 (bP)	Kappa A (bP)	Kappa B (bP)
Chronic lymphocytic leukemia/small lymphocytic lymphoma from 2018	340, 344	276, 828	140, 143	284	279
Diffuse large B-cell lymphoma germinal center phenotype (RT-1)	350	286	Negative	Negative	220, 290
Diffuse large B-cell lymphoma with nongerminal center phenotype (RT-2)	340–344	276, 828	140, 143	284	279

**Table 2:** A comparison of mutations identified by next generation sequencing for chronic lymphocytic leukemia/small lymphocytic lymphoma, clonally unrelated Richter transformation, and clonally related Richter transformation within the same patient.

Diagnosis	Associated mutation(s)			
	Gene	c	p	VAF (%)
Chronic lymphocytic leukemia/small lymphocytic lymphoma	*SF3B1*	c.1984C > T	p.H662Y	5

Diffuse large B-cell lymphoma, germinal center phenotype (RT-1)	*MYD88*	c.656C > G	p.S219C	3
	*TET2*	c.3405_3408dup	p.E1137cFs^∗^6	4
	*KMT2D*	c.10821_10823del	p.Q3612del	3

Diffuse large B-cell lymphoma with nongerminal center phenotype (RT-2)	*XPO1*	c.1612G > A	p.A538T	2
	*SF3B1*	c.1984C > T	p.H662Y	43
	*POT1*	c.820G > A	p.G274R	39
	*KRAS*	c.38G > A	p.G13D	44
	*PDS5B*	c.4169del	p.N1390Mfs^∗^4	5
	*TP53*	c.844C > G	p.S219C	81
	*BCORL1*	c.4860C > A	p.C1620^∗^	46

## Data Availability

Data that supports the findings of this study are provided within the manuscript, any additional data are available from the corresponding author upon reasonable request.
